# Association between dietary antioxidant intake and overweight/obesity risk among children and adolescents: a cross-sectional analysis from NHANES 2011–2016

**DOI:** 10.3389/fnut.2025.1540303

**Published:** 2025-05-06

**Authors:** Jinwen Chen, Changhong Shi

**Affiliations:** ^1^Department of Neonatology, Hefei Maternal and Child Health Hospital, Hefei, Anhui, China; ^2^School of Public Health, Guangzhou Medical University, Guangzhou, Guangdong, China

**Keywords:** composite dietary antioxidant index, obesity, children and adolescents, antioxidant intake, NHANES

## Abstract

**Objective:**

Overweight and obesity among children and adolescents has emerged as a critical global public health issue. Oxidative stress, a key factor in obesity-related inflammation and metabolic dysregulation, underscore the importance of dietary antioxidants. The composite dietary antioxidant index (CDAI), which integrates vitamins A, C, E, carotenoids, selenium, and zinc, provide a comprehensive measure of overall dietary antioxidant intake. However, the relationship between CDAI and overweight/obesity in children and adolescents remains insufficient explored.

**Methods:**

This study utilized data from the National Health and Nutrition Examination Survey (NHANES) collected between 2011 and 2016, including 17,919 participants aged 6–19 years. The CDAI were calculated based on dietary intake data from 24-hour dietary recalls. To account for total energy intake, two widely recognized adjustment methods were used: the standard regression model and the nutrient density model. In the nutrient density model, an energy-standardized CDAI (E-CDAI) was computed. Logistic regression models were conducted to examine associations between CDAI, E-CDAI, mCDAI, mE-CDAI, and overweight/obesity risk, adjusting for potential confounders such as age, gender, race, physical activity, and socioeconomic status.

**Results:**

The analysis showed a significant negative association between CDAI and overweight/obesity risk among adolescents aged 12–19 years. However, no significant association was observed in children aged 6–11 years. In contrast, E-CDAI showed no significant association with overweight/obesity risk in adolescents (OR = 0.87; 95% CI: 0.71–1.07). Notably, selenium exhibited a negative association with overweight/obesity in the standard regression model but a positive association in the nutrient density model. After excluding the selenium from the original 6 antioxidants included in the CDAI, the modified CDAI (mCDAI) demonstrated a significant negative association with overweight/obesity in both the standard regression model (OR = 0.74; 95% CI: 0.63–0.86) and nutrients density model (OR = 0.78; 95% CI: 0.69–0.89).

**Conclusion:**

This study developed a modified CDAI, comprising of vitamins A, C, E, carotenoids, and zinc, and identified a consistent negative association between mCDAI and overweight/obesity risk, irrespective of energy adjustment method. These findings suggest that a diet rich in antioxidants may play a protective role in preventing obesity in adolescent aged 12–19 years.

## 1 Introduction

Overweight and obesity have emerged as critical global public health challenges, particularly among children and adolescents. According to the latest World Health Organization (WHO) report, more than 300 million children and adolescents aged 5–19 years worldwide are classified as overweight or obese (1). The prevalence of overweight has increased nearly fivefold, while obesity rates have risen approximately sevenfold compared to levels four decades ago (2). In the United States, this trend is particularly pronounced, with the obesity rate among children and adolescent escalating from 17.7% in 2011 to 21.5% by 2020 (3). Obesity is now widely recognized as a complex, multifactorial disease that adversely impacts multiple physiological systems and can profoundly affect a child's intellectual, behavioral, psychological, and sexual development, with consequences that often persist throughout the lifespan (4, 5). Moreover, childhood obesity is strongly associated with an increased susceptibility to chronic condition in adulthood, including cardiovascular disease, type 2 diabetes mellitus (T2DM), non-alcoholic fatty liver disease (NAFLD) and certain types of cancer (6, 7). These compelling health implications underscore the urgent need for developing and implementing effective prevention and intervention strategies to mitigate the growing burden of childhood obesity.

Emerging evidence suggests that oxidative stress plays a pivotal role in the development and progression of obesity-related metabolic complications (8, 9). Excessive adipose tissue in obesity is a major source of reactive oxygen species (ROS), which can trigger chronic inflammation, contributing to insulin resistance, endothelial dysfunction, and other metabolic disturbances (10). These findings have sparked increasing interest in the potential protective effect of dietary antioxidants against obesity and its related disorders (11, 12). Some studies indicated that regions with high rates of antioxidant nutrient deficiencies also experience greater obesity prevalence (13). A recent systematic review found that obese individuals tend to have a lower concentration of antioxidants, particularly carotenoids, vitamins E and C, zinc, magnesium and selenium (14). However, contradictory results have been regarding the association between obesity and dietary antioxidants, especially in adolescents. For example, Galan et al. found no significant association between zinc and selenium concentration and obesity in 3,128 participants (15). Similarly, Yang et al. (16) found that selenium was not an independent protective factor against obesity in US adults but rather showed a positive association with obesity risk.

Although the health benefits of individual antioxidants have been extensively investigated, recent research has increasingly focused on the synergistic effects of multiple dietary antioxidants. Several studies have explored the relationship between weight status and various antioxidant indices, including the dietary antioxidant index (DAI) (17), total antioxidant capacity (TAC) (18), and dietary antioxidant quality score (DAQS)(19). Using weighted quantile sum (WQS) regression, Yang et al. (16) demonstrated that a combination of 11 antioxidants was negatively related to prevalence of obesity and abdominal obesity. The composite dietary antioxidant index (CDAI) is a comprehensive metric designed to assess overall dietary antioxidant intake by incorporating various key antioxidants (20), including vitamins A, C, E, carotenoids, selenium, and zinc. Previous studies have demonstrated an association between higher CDAI and reduced markers of oxidative stress and inflammation (21–24). Despite distinct dietary patterns and metabolic profiles in children and adolescents, the potential impact of overall antioxidant intake on obesity risk in this population remains underexplored.

This study aims to address this gap by examine the association between the composite dietary antioxidant index (CDAI) and the prevalence of overweight or obesity among children and adolescents in the United States, using data from the National Health and Nutrition Examination Survey (NHANES) from 2011 to 2016. We hypothesize that higher CDAI, indicative of greater antioxidant intake, is associated with a lower risk of overweight and obesity in this population. Total energy intake may represent a key confounder in the relationship between CDAI and overweight/obesity risk (25). To account for this, we employed two distinct models, the standard regression model and the nutrient density model (26). In the nutrient density model, we developed an energy-standardized CDAI (E-CDAI) score. Additionally, the dietary antioxidant quality score (DAQS) was calculated by comparing antioxidants intakes to their respective age-specified daily recommended intake values. Logistic regression models were conducted to examine associations between CDAI, E-CDAI, DAQS, and overweight/obesity risk, adjusting for potential confounders such as age, gender, race, physical activity, and socioeconomic status.

## 2 Methods

### 2.1 Study design and population

This study used data from the National Health and Nutrition Examination Survey (NHANES) conducted between 2011 and 2016. NHANES employs a complex, stratified, multistage probability sampling method to collect health and nutritional information from a representative sample of the civilian, non-institutionalized U.S. population. The survey protocol was approved by the NCHS Research Ethics Review Committee, and written informed consent was obtained from all participants. Detailed information on the NHANES design and procedures can be found in previous studies.

**Figure 1:**
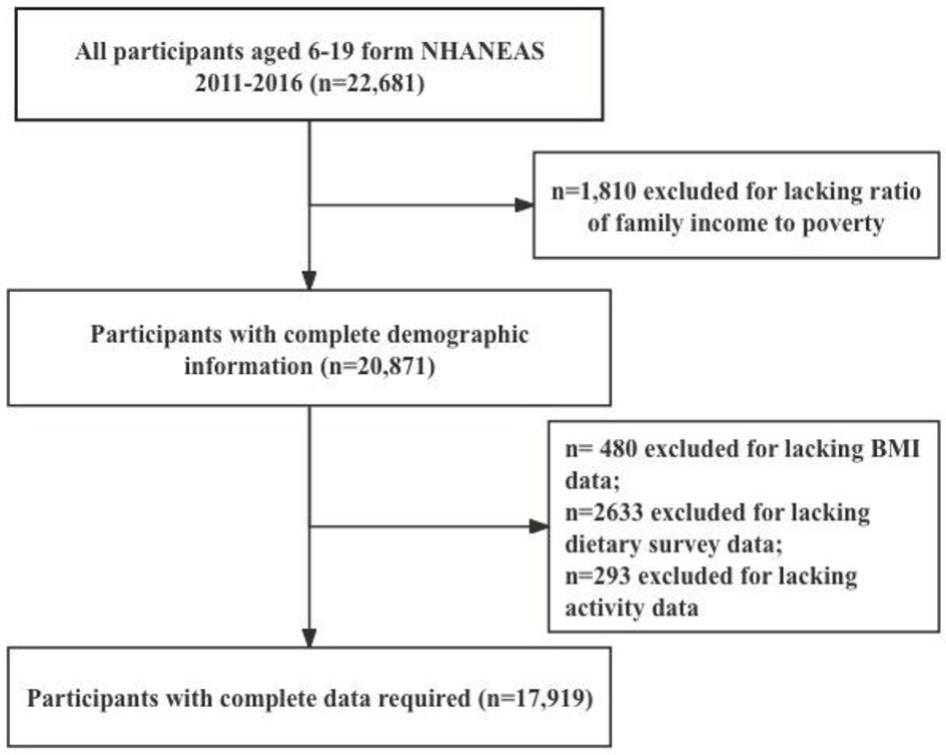
Flow chart of inclusion and exclusion criteria for the study sample. NHANES, National Health and Nutrition Survey; BMI, Body Mass Index.

Inclusion criteria required participants to have completed at least two 24-h dietary recalls. Exclusion criteria included missing data on key variables such as BMI, energy intake, or household income. The NHANES database provides information on various parameters such as age, gender, race, socioeconomic status, physical activity, energy intake, dietary components, and anthropometric measurements for study participants. This process resulted in a final sample of 17,919 participants. A detailed study flowchart is depicted in Figure 1.

### 2.2 Overweight and obesity data

Overweight and obesity data, including height and weight, were collected using standardized procedures. BMI was calculated as weight in kilograms divided by height in meters squared. Based on the CDC growth charts, participants were classified into the following categories (24): underweight (BMI below the 15th percentile), normal weight (BMI between the 15th and 85th percentiles), overweight (BMI between the 85th and 95th percentiles), and obesity (BMI at or above the 95th percentile).

### 2.3 Composite dietary antioxidant index

The composite dietary antioxidant index (CDAI) was computed using dietary data from the NHANES 24-h recall interviews. The index encompasses 6 key antioxidants: vitamins A, C, E, carotenoids, selenium, and zinc. To calculate the CDAI, we used the method proposed by Wright et al. (20), which involves standardizing each antioxidant by subtracting the global mean and dividing by the global standard deviation, which is calculated as follows:


$$\begin{array}{l} CDAI=\overset{6}{\underset{i=1}{\sum }}\frac{x_{i}-mean\left(x_{i}\right)}{std\left(x_{i}\right)}, \end{array}$$

where *x*_*i*_ represents the daily antioxidant intake, *mean*(*x*_*i*_) indicates the mean amount of these antioxidants within the study cohort, and *std*(*x*_*i*_) denotes the standard deviation. By leveraging these 24-h dietary recall data, CDAI scores can provide a comprehensive evaluation of the antioxidant intake at the individual level.

To adjust for total energy intake in the analysis, we employed two widely recognized adjustment approach, the standard regression model and the nutrient density model (26). Within the nutrient density model, we calculated the density of each antioxidant nutrient by dividing the absolute antioxidant intake by the total energy intake, expressed as *y*_*i*_ = *x*_*i*_/*energy*. Subsequently, we derived an energy-standardized CDAI (E-CDAI) using the following formula:


$$\begin{array}{l} E-CDAI=\overset{6}{\underset{i=1}{\sum }}\frac{y_{i}-mean\left(y_{i}\right)}{std\left(y_{i}\right)}. \end{array}$$

### 2.4 Covariates

NHANES adjusted for individual characteristics using several covariates, including age, sex, race, ethnicity, BMI, physical activity, poverty-income ratio and vitamin D. Detailed information on measurement procedures is available on the CDC website. Physical activity data were collected following World Health Organization guidelines. For adolescents, physical activity was categorized according to the 2018 Physical Activity Guidelines Advisory Committee report (27), participants who reported <10 min of moderate-to-vigorous physical activity per week were labeled as inactive. For children, physical activity was categorized into two groups: active (≥4 days) or inactive (<4 days) according to the question: “Days physically active at least 60 min.”

### 2.5 Statistical analysis

The study conducted descriptive analyses on the entire sample, with data further stratified by age group (children aged 6–11 years vs. adolescents aged 12–19 years). Results were presented as weighted median with interquartile range [median (interquartile range)] or percentage (%) for baseline characteristics. Continuous variables were analyzed using the Wilcoxon test or Kruskal–Wallis test. The association between categorical variables was examined using the Chi-square test. Multiple logistic regression was employed to assess the association between CDAI or E-CDAI and weighted-related measures (overweight/obesity, obesity). We construct 3 models to comprehensively evaluate the relationship between them: Model 1 includes age, gender, race, and household income-to-poverty ratio (PIR); Model 2 includes adjustments for physical activity. In model 3, we further adjusted for total energy intake. After dividing CDAI or E-CDAI into quartiles, trends tests were utilized to analyze their linear association trend. The results were presented as odds ratios (ORs) with 95% confidence intervals (CIs) across quartiles of CDAI and E-CDAI. All analyses were conducted using R software (version 4.4.2), with significance set at a *p*-value of < 0.05.

## 3 Results

### 3.1 Baseline characteristics

The study included 17,919 participants from the NHANES 2011–2016 dataset, consisting of 9,052 males (51.5%) and 8,867 females (48.5%). Participants were stratified into two age groups: children (6–11 years 43.1%) and adolescents (12–19 years, 56.9%). There were no statistically significant differences in gender, race/ethnicity, BMI status, energy intake, or poverty-income ratio between children and adolescents. However, adolescents had significantly lower average intakes of certain micronutrient, including vitamin D, vitamin A, vitamin C, and carotenoids, than children (*p* < 0.001). Conversely, selenium intake was higher in adolescents (*p* < 0.001). Overall, adolescents exhibited lower scores for both the composite dietary antioxidant index (CDAI) and the energy-standardized CDAI (E-CDAI) compared to children (CDAI: *p* = 0.025; E-CDAI: *p* < 0.001). These differences may reflect age-related changes in dietary habits, with adolescents possibly consuming more processed foods with lower antioxidant content. Table 1 details these baseline characteristics.

**Table 1:** Baseline characteristics of US children and adolescents aged 6–19 years, NHANES 2011–2016.^a^

**Characteristic**	**Overall**	**Children aged 6–11**	**Adolescents aged 12–19**	***P***
Overall, *n* (%)	17,919 (100%)	8,638 (43.1%)	9,281 (56.9%)	
Age (years)	12.00 (9.00, 16.00)	8.00 (7.00, 10.00)	16.00 (14.00, 17.00)	<0.001
**Gender (%)**				
Male	9,052.0 (51.5%)	4,372.0 (52.2%)	4,680.0 (51.0%)	0.6
Female	8,867.0 (48.5%)	4,266.0 (47.8%)	4,601.0 (49.0%)	
**Race (%)**				
Mexican American	3,722.0 (15.1%)	1,781.0 (15.4%)	1,941.0 (14.8%)	0.7
Other Hispanic	1,969.0 (7.5%)	980.0 (8.0%)	989.0 (7.2%)	
Non-Hispanic White	4,830.0 (54.0%)	2,387.0 (52.9%)	2,443.0 (54.8%)	
Non-Hispanic Black	4,629.0 (14.2%)	2,256.0 (14.1%)	2,373.0 (14.3%)	
Other race	2,769.0 (9.2%)	1,837.53 (9.5%)	1,535.0 (8.9%)	
Energy (kcal)	1,881.00 (1,534.00, 2,282.00)	1,859.39 (1,566.96, 2,208.00)	1,905.45 (1,489.00, 2,369.00)	0.077
**Physical activity (%)**				
Inactive	4,000.0 (22.0%)	1,282.0 (15.4%)	2,718.0 (27.0%)	<0.001
Active	13,919.0 (78.0%)	7,356.0 (84.6%)	6,563.0 (73.0%)	
**PIR, (%)**				
<130%	7,947.0 (34.3%)	3,970.0 (34.8%)	3,977.0 (33.8%)	0.7
>130%	9,972.0 (65.7%)	4,668.0 (65.2%)	5,304.0 (66.2%)	
**BMI status (%)**				
Underweight	578.0 (3.7%)	275.0 (4.6%)	303.0 (3.0%)	0.069
Normal weight	10,612.0 (60.2%)	5,216.0 (60.1%)	5,396.0 (60.2%)	
Overweight	3,038.0 (16.4%)	1,437.0 (17.1%)	1,601.0 (15.9%)	
Obesity	3,691.0 (19.7%)	1,710.0 (18.2%)	1,981.0 (20.8%)	
Vitamin D (mcg)	4.99 (2.92, 7.08)	5.66 (3.70, 7.55)	4.40 (2.40, 6.57)	<0.001
Vitamin A (mcg)	561.92 (379.00, 749.00)	617.00 (445.00, 780.08)	519.91 (332.00, 714.86)	<0.001
Vitamin C (mg)	60.30 (29.80, 95.12)	70.10 (39.32, 102.39)	53.10 (24.20, 88.89)	<0.001
Vitamin E (mg)	6.70 (4.91, 8.62)	6.79 (5.13, 8.47)	6.60 (4.73, 8.76)	0.2
Carotene (mcg)	828.00 (301.00, 1,443.00)	896.00 (321.00, 1,595.67)	780.84 (286.00, 1,355.05)	<0.001
Selenium (mcg)	96.90 (75.44, 121.42)	94.04 (75.08, 114.00)	100.07 (75.60, 129.40)	<0.001
Zinc (mg)	9.74 (7.39, 12.44)	9.75 (7.57, 11.91)	9.73 (7.24, 12.95)	0.4
CDAI	−0.54 (−2.14, 1.13)	−0.45 (−1.84, 1.02)	−0.67 (−2.35, 1.22)	0.025
E-CDAI	−0.71 (−1.78, 0.42)	−0.56 (−1.59, 0.48)	−0.82 (−1.94, 0.35)	0.001

BMI, Body Mass Index; PIR, income-to-poverty ratio; CDAI, Composite Dietary Antioxidant Index; E-CDAI, energy-standardized composite Dietary Antioxidant Index.

Data are presented as median [interquartile range], or n (%).

a All estimates are weighted except sample sizes (n).

### 3.2 Univariate analysis of overweight/obesity

As presented in Table 2, univariate analyses were conducted to evaluated factors associated with the risk of overweight/obesity. Ethnicity emerged as a significant factor, with Non-Hispanic White participants demonstrating the highest rates of overweight/obesity (*p* < 0.001). Lower income-to-poverty ratios were significant associated with a higher prevalence of overweight/obesity in both children (*p* = 0.023) and adolescents (*p* = 0.035). Among adolescents, overweight/obese individuals had significantly lower CDAI scores compared to their normal-weight counterparts (*p* < 0.001), whereas no significant differences were observed for the E-CDAI. In children, neither CDAI nor E-CDAI showed significant differences between BMI categories. Notably, overweight/obese adolescents had significant lower average intake of all assessed micronutrients, including vitamin D, vitamin A, vitamins C, vitamin E, carotene, selenium, and zinc (all *p* < 0.05). In contrast, no such difference were observed in children. These findings suggest that the role of dietary antioxidants in overweight/obesity may vary by age group, reflecting difference in metabolic profile and dietary patterns between children and adolescent.

**Table 2:** Univariate analysis of overweight/obesity in US children and adolescents aged 6–19 years, NHANES 2011–2016.^a^

**Characteristic**	**Children aged 6–11 years**			**Adolescents aged 12–19 years**		
	**Underweight/ normal** ***N*** = **5,491**	**Overweight/ obesity** ***N*** = **3,147**	***P***	**Underweight/ normal** ***N*** = **5,6991**	**Overweight/ obesity** ***N*** = **3,5821**	***P***
**Gender (%)**						
Male	2,779 (50.3%)	1,593 (55.7%)	0.10	2,934 (52.1%)	1,746 (49.2%)	0.3
Female	2,712 (49.7%)	1,554 (44.3%)		2,765 (47.9%)	1,836 (50.8%)	
**Race (%)**						
Mexican American	930 (12.3%)	851.0 (21.2%)	<0.001	1,049 (12.6%)	892 (18.7%)	<0.001
Other Hispanic	550 (6.8%)	430 (10.1%)		611 (7.0%)	378 (7.4%)	
Non-Hispanic White	1,671 (57.0%)	716 (45.5%)		1,586 (59.1%)	857 (47.5%)	
Non-Hispanic Black	1,443 (14.0%)	813 (14.5%)		1,384 (12.9%)	989 (16.6%)	
Other race	897.0 (9.9%)	337 (8.8%)		1,069 (8.4%)	466 (9.8%)	
Energy (kcal)	1,852.15 (1,563.00, 2,197.61)	1,874.68 (1,571.79, 2,232.00)	0.5	1,957.47 (1,550.00, 2,427.67)	1,812.60 (1,414.00, 2,256.00)	<0.001
**Physical activity (%)**						
Inactive	683 (11.6%)	599 (22.4%)	<0.001	1,599 (25.5%)	1,119 (29.6%)	0.15
Active	4,808 (88.4%)	2,548 (77.6%)		4,100 (74.5%)	2,463 (70.4%)	
**PIR (%)**						
<130%	2,400 (32.4%)	1,570 (39.3%)	0.023	2,300 (31.8%)	1,677 (37.4%)	0.035
>130%	3,091 (67.6%)	1,577 (60.7%)		3,399 (68.2%)	1,905 (62.6%)	
CDAI	−0.48 (−1.87, 0.95)	−0.38 (−1.81, 1.16)	0.4	−0.46 (−2.12, 1.52)	−1.05 (−2.74, 0.67)	<0.001
E-CDAI	−0.56 (−1.61, 0.49)	−0.56 (−1.54, 0.48)	0.8	−0.79 (−1.91, 0.40)	−0.90 (−2.04, 0.29)	0.3
Vitamin D (mcg)	5.60 (3.54, 7.56)	5.70 (3.81, 7.50)	0.5	4.50 (2.54, 6.80)	4.14 (2.20, 6.07)	<0.001
Vitamin A (mcg)	618.84 (444.00, 781.72)	614.25 (447.00, 777.00)	0.7	541.50 (359.00, 745.86)	483.32 (304.00, 663.18)	<0.001
Vitamin C (mg)	69.03 (38.51, 100.64)	72.30 (40.80, 105.20)	0.13	55.59 (26.70, 92.81)	48.66 (20.90, 82.50)	<0.001
Vitamin E (mg)	6.81 (5.14, 8.50)	6.74 (5.09, 8.44)	0.8	6.79 (4.92, 9.05)	6.34 (4.41, 8.30)	<0.001
Carotene (mcg)	905.40 (324.00, 1,654.00)	883.00 (316.00, 1,488.00)	0.3	801.00 (296.00, 1,389.97)	726.85 (269.00, 1,310.75)	0.027
Selenium (mcg)	92.72 (74.30, 112.40)	96.40 (77.00, 117.64)	0.015	102.64 (78.10, 133.30)	95.81 (72.10, 122.13)	<0.001
Zinc (mg)	9.67 (7.53, 11.89)	9.86 (7.59, 11.93)	0.5	10.04 (7.55, 13.38)	9.25 (6.78, 12.05)	<0.001

BMI, Body Mass Index; PIR, income-to-poverty ratio; CDAI, Composite Dietary Antioxidant Index; E-CDAI, energy-standardized composite Dietary Antioxidant Index.

Data are presented as median [interquartile range], or n (%).

a All estimates are weighted except sample sizes (n).

### 3.3 Association between CDAI and overweight/obesity

Logistic regression models were conducted to explore the relationship between the composite dietary antioxidant index (CDAI), energy-standardized CDAI (E-CDAI), and overweight/obesity risk across different age groups, as presented in Table 3.

**Table 3:** Association between overweight/obesity and CDAI, E-CDAI in children and adolescents in US, NHAENS 2011–2016.

**Subgroups**	**Q1**	**Q2 OR (95% CI)**	**Q3 OR (95% CI)**	**Q4 OR (95% CI)**	***P* for trends^d^**
**6–11, CDAI**					
Model 1^a^	Ref	1.03 (0.91, 1.17)	1.08 (0.95, 1.22)	1.06 (0.93, 1.20)	0.329
Model 2^b^	Ref	1.03 (0.90, 1.16)	1.08 (0.95, 1.22)	1.06 (0.93, 1.21)	0.285
Model 3^c^	Ref	1.01 (0.88, 1.15)	1.05 (0.91, 1.20)	1.01 (0.85, 1.19)	0.847
**6–11, E-CDAI**					
Model 1^a^	Ref	0.98 (0.86, 1.11)	1.06 (0.94, 1.20)	1.02 (0.90, 1.16)	0.577
Model 2^b^	Ref	0.98 (0.87, 1.12)	1.06 (0.94, 1.21)	1.01 (0.89, 1.15)	0.694
Model 3^c^	Ref	0.99 (0.87, 1.12)	1.07 (0.94, 1.21)	1.03 (0.90, 1.17)	0.515
**12–19, CDAI**					
Model 1^a^	Ref	0.85 (0.76, 0.96)	0.74 (0.657, 0.834)	0.576 (0.508, 0.652)	<0.001
Model 2^b^	Ref	0.86 (0.76, 0.96)	0.75 (0.66, 0.84)	0.58 (0.51, 0.66)	<0.001
Model 3^c^	Ref	0.93 (0.82, 1.05)	0.84 (0.74, 0.96)	0.73 (0.61, 0.86)	<0.001
**12–19, E-CDAI**					
Model 1^a^	Ref	1.00 (0.89, 1.13)	0.99 (0.88, 1.12)	0.94 (0.83, 1.06)	0.295
Model 2^b^	Ref	1.01 (0.90, 1.14)	1.00 (0.88, 1.12)	0.94 (0.84, 1.07)	0.318
Model 3^c^	Ref	1.00 (0.89, 1.13)	0.96 (0.85, 1.08)	0.86 (0.76, 1.07)	0.061

OR, odds ratio; 95% CI, 95% confidence interval.

a Model 1, adjusted for age, gender, race and household income-to-poverty ratio (PIR).

b Model 2, adjusted for covariates of model 1 plus physical activity.

c Model 3, according to model 2 plus the total energy intake.

d *P* for trend based on variable containing median value for each quantile.

In children aged 6–11 years, no statistically significant associations were observed between either CDAI or E-CDAI and overweight/obesity in children across all adjusted models. The odds ratios (ORs) for overweight/obesity show no significant variation across quartiles of CDAI (*p* for trend > 0.05). Similarly, E-CDAI also demonstrated no significant association with overweight/obesity in this age group (*p* for trend > 0.05).

Among adolescents aged 12–19 years, a significant negative association was observed between CDAI and the risk of overweight/obesity among adolescents. Participants in the highest quartile of CDAI had a significantly lower odds of being overweight/obese compared to those in the lowest quartile (Model 3 OR = 0.73; 95% CI: 0.61–0.86; *p* for trend < 0.001). However, in the nutrient density model, E-CDAI showed no significant association with overweight/obesity risk (*p* for trend = 0.061). This discrepancy suggest that the method of energy adjustment may significantly influence the observed association between antioxidants indices and overweight/obesity.

### 3.4 Association of antioxidants with overweight/obesity

We explored the relationship between overweight/obesity and both the absolute intake and nutrient density of individual antioxidants included in the CDAI, such as carotenoids, vitamins E and C, zinc, magnesium, and selenium. Comparative analyses of these measures are presented in Tables 4, 5. Nutrient density for each antioxidant nutrient was calculated as the ratio of absolute antioxidant intake to total energy intake.

**Table 4:** Association between overweight/obesity and individual antioxidants in children and adolescents.

**Variables**	**Children aged 6–11 years**			**Adolescents aged 12–19 years**		
	**Model 1** ^a^	**Model 2** ^b^	**Model 3** ^c^	**Model 1** ^a^	**Model 2** ^b^	**Model 3** ^c^
**Vitamin A**						
Q2	1.04 (0.91, 1.18)	1.04 (0.92, 1.18)	1.03 (0.9, 1.17)	0.97 (0.86, 1.09)	0.97 (0.86, 1.09)	1.02 (0.91, 1.15)
Q3	1.09 (0.96, 1.23)	1.08 (0.95, 1.23)	1.06 (0.93, 1.21)	0.75 (0.67, 0.85)^*^	0.76 (0.67, 0.85)^*^	0.83 (0.74, 0.94)^*^
Q4	1.04 (0.91, 1.18)	1.03 (0.91, 1.17)	0.99 (0.86, 1.14)	0.61 (0.54, 0.69)^*^	0.62 (0.54, 0.7)^*^	0.73 (0.63, 0.83)^*^
**Vitamin C**						
Q2	0.92 (0.81, 1.04)	0.92 (0.81, 1.05)	0.91 (0.8, 1.04)	0.88 (0.78, 0.99)^*^	0.88 (0.78, 0.99)^*^	0.93 (0.82, 1.04)
Q3	0.97 (0.86, 1.1)	0.98 (0.86, 1.11)	0.96 (0.85, 1.09)	0.79 (0.7, 0.89)^*^	0.79 (0.7, 0.89)^*^	0.85 (0.75, 0.96)^*^
Q4	0.91 (0.8, 1.04)	0.92 (0.81, 1.05)	0.9 (0.79, 1.03)	0.7 (0.62, 0.79)^*^	0.7 (0.62, 0.79)^*^	0.8 (0.7, 0.9)^*^
**Vitamin E**						
Q2	0.99 (0.87, 1.12)	0.99 (0.88, 1.13)	0.96 (0.84, 1.09)	0.83 (0.73, 0.93)^*^	0.83 (0.74, 0.93)^*^	0.91 (0.81, 1.03)
Q3	0.89 (0.78, 1.01)	0.89 (0.78, 1.01)	0.84 (0.73, 0.96)	0.77 (0.68, 0.87)^*^	0.77 (0.68, 0.87)^*^	0.9 (0.79, 1.03)
Q4	0.96 (0.85, 1.09)	0.98 (0.86, 1.11)	0.88 (0.75, 1.03)	0.64 (0.57, 0.73)^*^	0.65 (0.57, 0.73)^*^	0.85 (0.73, 1)
**Carotene**						
Q2	0.93 (0.82, 1.06)	0.93 (0.82, 1.06)	0.91 (0.8, 1.04)	1.01 (0.9, 1.14)	1.01 (0.9, 1.14)	1.12 (0.99, 1.26)
Q3	1.05 (0.92, 1.19)	1.05 (0.92, 1.19)	1.03 (0.91, 1.17)	0.9 (0.8, 1.01)	0.9 (0.8, 1.01)	1.01 (0.89, 1.14)
Q4	0.89 (0.78, 1.01)	0.9 (0.79, 1.02)	0.88 (0.77, 1)	0.81 (0.72, 0.91)^*^	0.82 (0.72, 0.92)^*^	0.93 (0.82, 1.06)
**Selenium**						
Q2	1.01 (0.89, 1.15)	1.01 (0.89, 1.15)	1.02 (0.89, 1.16)	0.88 (0.78, 0.99)^*^	0.88 (0.78, 0.99)^*^	0.99 (0.88, 1.12)
Q3	1.11 (0.98, 1.26)	1.11 (0.98, 1.26)	1.12 (0.98, 1.29)	0.78 (0.69, 0.88)^*^	0.79 (0.7, 0.89)^*^	0.96 (0.84, 1.09)
Q4	1.16 (1.02, 1.32)	1.17 (1.03, 1.33)	1.19 (1.01, 1.4)	0.7 (0.62, 0.8)^*^	0.71 (0.62, 0.8)^*^	1.02 (0.86, 1.2)
**Zinc**						
Q2	0.97 (0.85, 1.1)	0.97 (0.85, 1.1)	0.96 (0.84, 1.09)	0.88 (0.78, 0.99)^*^	0.89 (0.79, 1)^*^	0.99 (0.87, 1.11)
Q3	1.08 (0.95, 1.23)	1.08 (0.95, 1.23)	1.06 (0.92, 1.22)	0.84 (0.74, 0.94)^*^	0.84 (0.75, 0.95)^*^	1 (0.88, 1.14)
Q4	1.08 (0.95, 1.23)	1.09 (0.96, 1.23)	1.06 (0.9, 1.23)	0.67 (0.59, 0.76)^*^	0.67 (0.59, 0.77)^*^	0.91 (0.78, 1.06)

Data are presented as OR (95% CI), OR, odds ratio; 95% CI, 95% Confidence Interval.

a Model 1, adjusted for age, gender, race and household income-to-poverty ratio (PIR).

b Model 2, adjusted for covariates of model 1 plus physical activity.

c Model 3, according to model 2 plus the total energy intake.

* denotes *P* < 0.05.

**Table 5:** Association between overweight/obesity and antioxidants^d^ density in children and adolescents.

**Variables**	**Children aged 6–11 years**			**Adolescents aged 12–19 years**		
	**Model 1** ^a^	**Model 2** ^b^	**Model 3** ^c^	**Model 1** ^a^	**Model 2** ^b^	**Model 3** ^c^
**Vitamin A**						
Q2	1.01 (0.89, 1.14)	1 (0.88, 1.14)	1 (0.88, 1.13)	0.97 (0.86, 1.09)	0.97 (0.86, 1.09)	0.99 (0.88, 1.11)
Q3	1.02 (0.9, 1.16)	1.01 (0.89, 1.15)	1.01 (0.89, 1.15)	0.89 (0.79, 1)^*^	0.89 (0.79, 1)^*^	0.89 (0.79, 1)^*^
Q4	1.01 (0.89, 1.14)	0.99 (0.87, 1.13)	1 (0.88, 1.14)	0.79 (0.7, 0.89)^*^	0.79 (0.7, 0.89)^*^	0.76 (0.68, 0.86)^*^
**Vitamin C**						
Q2	0.9 (0.79, 1.02)	0.9 (0.79, 1.02)	0.9 (0.79, 1.02)	0.9 (0.8, 1.01)	0.9 (0.8, 1.01)	0.93 (0.82, 1.04)
Q3	0.92 (0.81, 1.04)	0.92 (0.81, 1.05)	0.92 (0.81, 1.04)	0.82 (0.73, 0.93)^*^	0.83 (0.73, 0.93)^*^	0.84 (0.74, 0.94)^*^
Q4	0.96 (0.85, 1.09)	0.97 (0.85, 1.1)	0.98 (0.86, 1.11)	0.86 (0.76, 0.97)^*^	0.86 (0.76, 0.97)^*^	0.84 (0.75, 0.95)^*^
**Vitamin E**						
Q2	1.03 (0.91, 1.17)	1.03 (0.91, 1.17)	1.03 (0.91, 1.17)	0.91 (0.81, 1.02)	0.91 (0.81, 1.02)	0.95 (0.84, 1.07)
Q3	1 (0.88, 1.14)	1 (0.88, 1.14)	1 (0.88, 1.13)	0.85 (0.75, 0.95)^*^	0.85 (0.75, 0.95)^*^	0.87 (0.77, 0.98)^*^
Q4	0.94 (0.83, 1.07)	0.94 (0.83, 1.07)	0.94 (0.83, 1.07)	0.88 (0.78, 0.99)^*^	0.88 (0.78, 0.99)^*^	0.9 (0.8, 1.01)
**Carotene**						
Q2	0.94 (0.83, 1.07)	0.94 (0.83, 1.07)	0.94 (0.83, 1.07)	1.09 (0.97, 1.23)	1.1 (0.97, 1.23)	1.14 (1.01, 1.29)
Q3	0.96 (0.85, 1.09)	0.96 (0.84, 1.09)	0.96 (0.84, 1.09)	1.05 (0.93, 1.18)	1.05 (0.93, 1.18)	1.07 (0.95, 1.21)
Q4	0.9 (0.79, 1.02)	0.91 (0.8, 1.03)	0.91 (0.8, 1.03)	0.92 (0.82, 1.04)	0.93 (0.82, 1.05)	0.93 (0.82, 1.05)
**Selenium**						
Q2	0.99 (0.87, 1.13)	1 (0.88, 1.14)	1 (0.88, 1.13)	1.06 (0.94, 1.19)	1.06 (0.94, 1.19)	1.06 (0.94, 1.2)
Q3	1.14 (1, 1.29)	1.13 (1, 1.29)	1.14 (1, 1.29)	1.1 (0.98, 1.24)	1.1 (0.97, 1.24)	1.07 (0.95, 1.21)
Q4	1.13 (0.99, 1.28)	1.12 (0.99, 1.28)	1.13 (1, 1.29)	1.24 (1.1, 1.39)^*^	1.23 (1.09, 1.39)^*^	1.15 (1.01, 1.29)^*^
**Zinc**						
Q2	1.05 (0.93, 1.19)	1.05 (0.93, 1.2)	1.05 (0.93, 1.2)	1 (0.89, 1.13)	1 (0.89, 1.13)	1.01 (0.89, 1.13)
Q3	1.02 (0.9, 1.16)	1.02 (0.9, 1.16)	1.03 (0.91, 1.17)	1.08 (0.96, 1.22)	1.08 (0.96, 1.22)	1.06 (0.94, 1.2)
Q4	1.05 (0.92, 1.19)	1.04 (0.92, 1.18)	1.05 (0.93, 1.19)	0.97 (0.86, 1.1)	0.98 (0.87, 1.1)	0.92 (0.82, 1.04)

Data are presented as OR (95% CI), OR, odds ratio; 95% CI, 95% confidence interval.

a Model 1, adjusted for age, gender, race and household income-to-poverty ratio (PIR).

b Model 2, adjusted for covariates of model 1 plus physical activity.

c Model 3, according to model 2 plus the total energy intake.

d Energy-standardized nutrient intake is defined as the ratio between antioxidants and total energy intake.

* denotes *P* < 0.05.

In children aged 6–11 years, no significant associations were found between the intake of individual antioxidants (vitamins A, C, E, carotenoids, selenium, and zinc) and the risk of overweight/obesity in children after adjustment for potential confounding factors.

Among adolescents aged 12–19 years, higher intakes of vitamins A, C, and E were significantly associated with a reduced risk of overweight/obesity in adolescents. Specifically, individual in the highest quartile of vitamin A intake had a 27% lower risk of overweight/obese compared to those in the lowest quartile (Model 3 OR = 0.73; 95% CI: 0.63–0.83). A similar protective effect was observed for vitamin C intake (Model 3 OR = 0.80; 95% CI: 0.70–0.90). Notably, these reverse association remained statistically significant in the nutrient density model (Table 6). Interestingly, while selenium density showed a positive association with overweight/obese (Model 2 OR = 1.23; 95% CI: 1.09–1.39; Table 5), absolute selenium showed negative association (Model 2 OR = 0.71, 95% CI: 0.62–0.80) (Table 4), suggesting a complex relationship between selenium and weight status.

**Table 6:** Association between overweight/obesity and mCDAI, mE-CDAI in children and adolescents in US, NHAENS 2011–2016.

**Subgroups**	**Q1**	**Q2 OR (95% CI)**	**Q3 OR (95% CI)**	**Q4 OR (95% CI)**	***P* for trends^d^**
**6–11, mCDAI**					
Model 1^a^	Ref	1.02 (0.9, 1.16)	1.04 (0.91, 1.18)	0.98 (0.87, 1.12)	0.809
Model 2^b^	Ref	1.02 (0.9, 1.16)	1.04 (0.92, 1.18)	0.99 (0.87, 1.13)	0.899
Model 3^c^	Ref	0.99 (0.87, 1.13)	0.99 (0.86, 1.13)	0.91 (0.78, 1.06)	0.235
**6–11, mE-CDAI**					
Model 1^a^	Ref	0.89 (0.78, 1.01)	0.95 (0.84, 1.08)	0.96 (0.84, 1.08)	0.716
Model 2^b^	Ref	0.89 (0.79, 1.01)	0.95 (0.84, 1.08)	0.95 (0.84, 1.08)	0.629
Model 3^c^	Ref	0.89 (0.79, 1.01)	0.96 (0.84, 1.09)	0.96 (0.85, 1.09)	0.785
**12–19, mCDAI**					
Model 1^a^	Ref	0.89 (0.79, 1)	0.74 (0.66, 0.83)	0.59 (0.52, 0.67)	<0.001
Model 2^b^	Ref	0.89 (0.79, 1)	0.74 (0.66, 0.84)	0.59 (0.53, 0.67)	<0.001
Model 3^c^	Ref	0.96 (0.85, 1.08)	0.84 (0.74, 0.96)	0.74 (0.63, 0.86)	<0.001
**12–19, mE-CDAI**					
Model 1^a^	Ref	0.89 (0.79, 1)	0.87 (0.77, 0.98)	0.83 (0.74, 0.94)	0.003
Model 2^b^	Ref	0.89 (0.79, 1)	0.87 (0.77, 0.98)	0.83 (0.74, 0.94)	0.004
Model 3^c^	Ref	0.9 (0.8, 1.01)	0.85 (0.76, 0.96)	0.78 (0.69, 0.89)	<0.001

OR, odds ratio; 95% CI, 95% confidence interval.

a Model 1, adjusted for age, gender, race and household income-to-poverty ratio (PIR).

b Model 2, adjusted for covariates of model 1 plus physical activity.

c Model 3, according to model 2 plus the total energy intake.

d *P* for trend based on variable containing median value for each quantile.

### 3.5 Association between modified CDAI and overweight/obesity

The above analysis demonstrated that the method of energy adjustment can reverse the direction of the association between selenium and weight status, potentially explaining the lack of a significant association between E-CDAI and overweight/obesity was observed in the multivariate nutrient density model. To address this, we developed a modified CDAI (mCDAI) score by excluding selenium from the original 6 antioxidants included in the CDAI. Following the same analytical apporach, we applied both the standard regression model and nutrient density model to adjust the confounding effect of total energy intake. Logistic regression models were conducted to explore the relationship between the modified composite dietary antioxidant index (mCDAI), modified energy-standardized CDAI (mE-CDAI), and overweight/obesity risk across different age groups, as detailed in Table 6.

In children aged 6–11 years, no statistically significant associations were observed between either mCDAI or mE-CDAI and overweight/obesity in children across all adjusted models. In contrast, among adolescents aged 12–19 years, a significant negative association was observed between mCDAI and the risk of overweight/obesity among adolescents in both standard regression model (Model 3 OR = 0.74; 95% CI: 0.63–0.86; *p* for trend < 0.001) and multivariate nutrient density model (Model 3 OR = 0.78; 95% CI: 0.69–0.89; *p* for trend < 0.001). These findings suggest that the exclusion of selenium from the CDAI may enhance the robustness of the association between dietary intake and overweight/obesity. In addition, there was no sex difference in the relationship between mCDAI, mE-CDAI and overweight/obesity (Table 7).

**Table 7:** Association between obesity and mCDAI, mE-CDAI in adolescents aged 12–19 years by sex NHAENS 2011–2016.

**Subgroups**	**Q1**	**Q2 OR (95% CI)**	**Q3 OR (95% CI)**	**Q4 OR (95% CI)**	***P* for trends^d^**
**Male, mCDAI**					
Model 1^a^	Ref	0.8 (0.68, 0.94)	0.72 (0.61, 0.85)	0.55 (0.46, 0.65)	<0.001
Model 2^b^	Ref	0.81 (0.69, 0.96)	0.74 (0.63, 0.88)	0.56 (0.47, 0.67)	<0.001
Model 3^c^	Ref	0.89 (0.75, 1.06)	0.87 (0.72, 1.05)	0.73 (0.59, 0.91)	0.007
**Male, mE-CDAI**					
Model 1^a^	Ref	0.87 (0.74, 1.03)	0.92 (0.78, 1.09)	0.83 (0.7, 0.98)	0.05
Model 2^b^	Ref	0.88 (0.74, 1.04)	0.93 (0.78, 1.1)	0.84 (0.71, 1)	0.078
Model 3^c^	Ref	0.88 (0.74, 1.04)	0.91 (0.77, 1.08)	0.79 (0.66, 0.94)	0.011
**Female, mCDAI**					
Model 1^a^	Ref	0.94 (0.8, 1.11)	0.81 (0.68, 0.96)	0.59 (0.49, 0.7)	<0.001
Model 2^b^	Ref	0.94 (0.8, 1.11)	0.81 (0.68, 0.95)	0.58 (0.49, 0.69)	<0.001
Model 3^c^	Ref	0.99 (0.83, 1.18)	0.87 (0.72, 1.05)	0.66 (0.53, 0.82)	<0.001
**Female, mE-CDAI**					
Model 1^a^	Ref	0.93 (0.79, 1.1)	0.87 (0.73, 1.03)	0.84 (0.71, 1)	0.036
Model 2^b^	Ref	0.93 (0.79, 1.1)	0.86 (0.73, 1.02)	0.84 (0.71, 1)	0.033
Model 3^c^	Ref	0.93 (0.79, 1.11)	0.84 (0.71, 1)	0.79 (0.67, 0.94)	0.004

OR, odds ratio; 95% CI, 95% confidence interval.

a Model 1, adjusted for age, gender, race and household income-to-poverty ratio (PIR).

b Model 2, adjusted for covariates of model 1 plus physical activity.

c Model 3, according to model 2 plus the total energy intake.

d *P* for trend based on variable containing median value for each quintile.

### 3.6 Association of DAQS with overweight/obesity

Recognizing that nutritional requirement vary significantly across different developmental stages, particularly within the broad age range of 6–19 years, we evaluated individual antioxidant against the daily recommended intake (DRI) established by the National Academies of Science, Engineering, and Medicine (NASEM) (28). Given the absence of an official DRI for carotenoids, this nutrient was excluded from the analysis. For each of five remained nutrients, a binary scoring system was implemented: a score of 0 was assigned if the nutrient intake fell below the age-specific DRI, and a score of 1was assigned if the intake meets or exceeded the age-specific DRI. Subsequently, we calculated the dietary antioxidant quality score (DAQS) by summing the score for the five antioxidants, resulting in a scale ranging from 0 (very poor quality) to 5 (high quality).

We further examined the association between overweight/obesity and both individual antioxidant score and the DAQS, as presented in Table 8. In children aged 6–11 years, no statistically significant associations were observed between overweight/obesity and either DAQS or individual antioxidant across all adjusted models. In contrast, among adolescents aged 12–19 years, a significant negative association was observed between DAQS and the risk of overweight/obesity (OR = 0.91; 95% CI: 0.86–0.96). Additionally, vitamins A (OR = 0.83; 95% CI: 0.73–0.94) and vitamins C (OR = 0.88; 95% CI: 0.86–0.96) demonstrated significant inverse association with overweight/obesity in this age group. However, no significant associations were observed between overweight/obesity and Vitamin E, Selenium or Zinc in adolescents.

**Table 8:** Association between overweight/obesity and DAQS and individual antioxidants score in children and adolescents.

**Variables**	**Children aged 6–11 years**			**Adolescents aged 12–19 years**		
	**Model 1** ^a^	**Model 2** ^b^	**Model 3** ^c^	**Model 1** ^a^	**Model 2** ^b^	**Model 3** ^c^
Vitamin A	0.99 (0.89, 1.11)	0.99 (0.89, 1.11)	0.96 (0.85, 1.08)	0.71 (0.63, 0.8) ^*^	0.72 (0.64, 0.81) ^*^	0.83 (0.73, 0.94) ^*^
Vitamin C	1 (0.92, 1.1)	1.01 (0.92, 1.11)	1 (0.91, 1.1)	0.82 (0.75, 0.89) ^*^	0.82 (0.75, 0.9) ^*^	0.88 (0.8, 0.96) ^*^
Vitamin E	0.92 (0.83, 1.02)	0.92 (0.84, 1.02)	0.89 (0.81, 0.99) ^*^	0.91 (0.83, 1.01)	0.92 (0.83, 1.01)	1.01 (0.91, 1.12)
Selenium	1.02 (0.88, 1.18)	1.02 (0.88, 1.19)	0.98 (0.83, 1.15)	0.79 (0.69, 0.9) ^*^	0.79 (0.7, 0.9) ^*^	1 (0.87, 1.15)
Zinc	1.2 (0.95, 1.52)	1.2 (0.95, 1.52)	1.15 (0.91, 1.47)	0.75 (0.62, 0.91) ^*^	0.75 (0.62, 0.91) ^*^	0.89 (0.73, 1.09)
DAQS	0.98 (0.93, 1.03)	0.99 (0.94, 1.04)	0.96 (0.91, 1.02)	0.86 (0.82, 0.91) ^*^	0.87 (0.82, 0.91) ^*^	0.91 (0.86, 0.96) ^*^

Data are presented as OR (95% CI), OR, odds ratio; 95% CI, 95% confidence Interval.

a Model 1, adjusted for age, gender, race and household income-to-poverty ratio (PIR).

b Model 2, adjusted for covariates of model 1 plus physical activity.

c Model 3, according to model 2 plus the total energy intake.

* denotes *P* < 0.05.

## 4 Discussion

In this cross-sectional study, we investigate the relationship between the composite dietary antioxidant index (CDAI) and overweight/obesity among 17,919 participants aged 6–19 years in the United States, utilizing data from NHANES spanning 2011–2016. We employed two distinct models, the standard regression model and the nutrient density model, to adjust for total energy intake. In the standard regression model, a significant negative association was observed between CDAI score and overweight/ obesity among adolescents, but not among children. Similarly, the dietary antioxidant quality score (DAQS) was also inversely associated with overweight/obesity in adolescents. However, in the nutrient density model, no significant association was found between the energy standardized CDAI (E-CDAI) score and overweight/obesity in either age group. Interestingly, we find that selenium showed a negative association with overweight/obesity in the standard regression model, but a positive association in the nutrient density model. After excluding the selenium from the original 6 antioxidants included in the CDAI, the modified CDAI (mCDAI) demonstrated a significant negative association with overweight/obesity in both the standard regression model.

Oxidative stress plays a critical role in the pathogenesis of obesity (29), characterized by an imbalance between reactive oxygen species (ROS) production and the body's antioxidant defenses. Increased adiposity is linked with heightened ROS production, leading to oxidative damage and chronic inflammation (30), which can further exacerbate fat accumulation and metabolic dysregulation. Numerous studies have investigated the association between individual antioxidants and obesity, as well as the underlying causal mechanism. For instance, Aeberli et al. (31) found that dietary intake of antioxidant vitamins (vitamin E, A, and C) was significantly associated with leptin level in Swedish children, suggesting that low concentration of these vitamins may alter the leptin genetic expression, contributing to leptin resistance and increases obesity risk. Similarly, Puchau et al. (18) reported that obese children and adolescents consumed lower amounts of vitamin E and C compared to their non-obese counterparts. A case-control study in Thailand further identified a negative association between BMI, waist, and serum concentrations of vitamins E (32). These findings align with our partial results, which indicate that vitamins A, C, and E were inversely associated with overweight/obesity in adolescents, both in the standard regression model and nutrient density model. Previous research has also demonstrated significant associations between dietary carotene intake and blood carotenoid levels, with lower serum carotene concentrations often observed in obese individuals (33). Our analysis similarly revealed a negative association between absolute carotene intake and obesity risk; however, this association became non-significant in the nutrient density model. Interestingly, selenium exhibits a negative association with obesity risk in adolescents in the standard regression model, but this relationship shifted to a positive association in the nutrient density model. Some studies have reported a significant correlation between dietary selenium intake and obesity. For example, Yang et al. (16) found that selenium was not an independent protective factor against obesity in US adults but was positive associated with it. Emerging evidence suggests that selenium toxicity can manifests as oxidative stress, impaired biofilm development, and suppression of enzyme function when present in excessive amounts (34, 35). These findings are consistent with our results.

Given that the effect of antioxidants is often dependent on their interaction with one another, their collective effect on weight status may differ from that of individual antioxidant. Therefore, in this study we focus on the association between the composite dietary antioxidant index (CDAI) and overweight/obesity in children and adolescents. To our knowledge, few studies have explored similar relationships using alternative dietary antioxidant indices. Notably, Kokkou et al. conducted a cross-sectional study involving 1,580 students aged 10–12 years, utilizing a dietary antioxidant index (DAI) that incorporated magnesium instead of carotenoids (17). Their findings revealed a significant inverse correlation between DAI scores and body weight status. Similarly, Aminnejad et al. investigated a modified version of DAI (including manganese rather than carotenoids) in a cohort of 593 adolescent boys (12–16 years), demonstrating a beneficial association between elevated antioxidant intake and improved weight status (36). In contrast, a study employing the dietary antioxidant quality score (DAQS) (excluding magnesium) in a larger sample of 4,270 participants aged 6–18 years paradoxically found that overweight and obese children exhibited higher intakes of certain dietary antioxidants compared to their normal-weight counterparts (19). These findings provide valuable insights for further research and underscore the need for standardized assessment of dietary antioxidant capacity in future studies.

**Figure 2:**
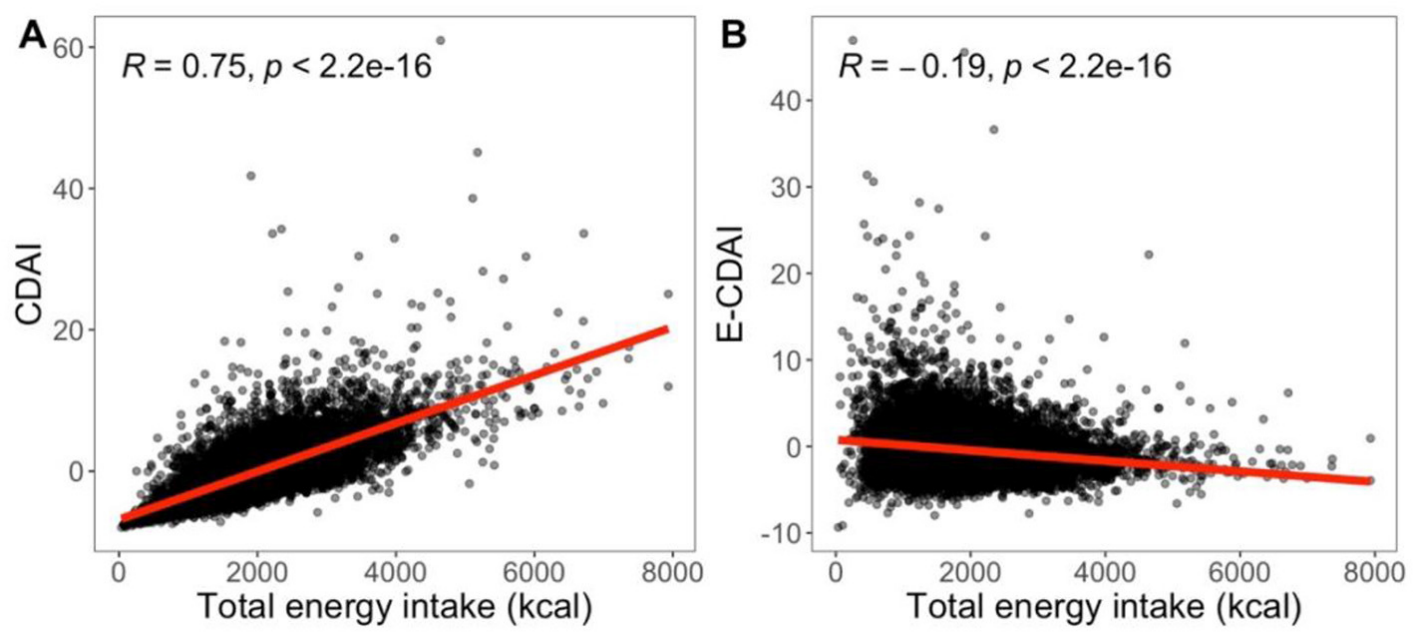
**(A)** CDAI has a high positive correlation with total energy intake (*r* = 0.75); **(B)** E-CDAI has a weak negative correlation with total energy intake (*r* = −0.19). Both were evaluated using the Spearman coefficient.

Nutrient intake is generally correlated with total energy intake, as individuals who consume more energy tend to ingest larger quantities of most specific nutrients. As depicted in Figure 2A, CDAI shows a strong positive correlation with total energy intake. Total energy intake is a well-established risk factor for overweight/obesity, contributing to increased adiposity regardless of the dietary composition (37). Thus, total energy intake may represent a key confounder in the relationship between CDAI and overweight/obesity risk. To adjust for the total energy intake, we employed both the standard regression model and the nutrient density model (E-CDAI). As illustrated in Figure 2B, E-CDAI displayed a weak negative correlation with total energy intake. Our findings indicate a significant negative association between CDAI and the risk of overweight/obesity among adolescents, whereas no significant relationship was observed between E-CDAI and overweight/obesity in either age group. Further analysis revealed a negative association between overweight/obesity and absolute selenium intake, but a positive association between overweight/obesity and selenium density, potentially explaining the lack of a significant association between E-CDAI and overweight/obesity was observed in the nutrient density model. To address this, we developed a modified CDAI (mCDAI) score by excluding selenium from the original 6 antioxidants included in the CDAI. Further analysis demonstrated the modified CDAI (mCDAI) showed a significant negative association with overweight/obesity in both the standard regression model. Since Willet and Stampfer's 1986 publication (38), most nutritional epidemiology studies have routinely incorporated some form of energy adjustment. However, debate persist regarding the most appropriate approach (39, 40). The two models differ in their interpretation: in the standard regression model, the regression coefficient represents the apparent effect of increasing the antioxidant by 1 unit while maintaining a constant total energy intake; in the multivariate nutrient density model, the regression coefficients reflect the apparent effect of nutrient density in units of the percentage of energy from the nutrient. Given of the longstanding use of nutrient densities by nutritionists and the application of nutrient densities in public health recommendations (41, 42), the nutrient density model is widely adopted for estimating dietary effects.

There are several limitations to this study that warrant consideration. The cross-sectional design prevents us from inferring causality between CDAI and obesity. Longitudinal studies are needed to explore whether increasing energy-standardized antioxidant intake can help prevent obesity over time. Additionally, the reliance on self-reported dietary recalls may introduce bias and inaccuracies in estimating antioxidant intake and energy consumption. Using objective biomarkers of antioxidant status and energy expenditure in future studies could provide more reliable data. Furthermore, this study did not account for other factors that could influence the relationship between antioxidants and obesity, such as individual genetics, hormonal status and gut microbiota. Future research should explore these factors to better understand the complex interactions between diet, oxidative stress, and obesity risk.

## 5 Conclusion

In conclusion, this study found that higher absolute dietary antioxidant intake, as measured by the composite dietary antioxidant index (CDAI), was significantly associated with a reduced risk of overweight and obesity among adolescents aged 12–19 years. However, the energy-standardized CDAI (E-CDAI) did not show a significant relationship with overweight/obesity. When selenium was excluded from the original CDAI, the modified CDAI (mCDAI) demonstrated a robust negative association with overweight/obesity in adolescents, irrespective of energy adjustment method. These findings suggest that a diet rich in antioxidants may play a protective role in preventing obesity in adolescent aged 12–19 years. Further longitudinal studies are needed to validate these findings and to elucidate the underlying mechanisms.

## Data Availability

Publicly available datasets were analyzed in this study. This data can be found at: http://www.cdc.gov/nchs/nhanes.htm.
